# Supplementary material for: Luminal and Tumor-Associated Gut Microbiome Features Linked to Precancerous Lesions Malignancy Risk: A Compositional Approach
Source: Cancers (Basel). 2022 Oct 24;14(21):5207. doi: 10.3390/cancers14215207 (PMC9653741; doi:10.3390/cancers14215207)
Supplement: Supplementary file 1 [file cancers-14-05207-s001.zip › Supplementary figures.pdf]

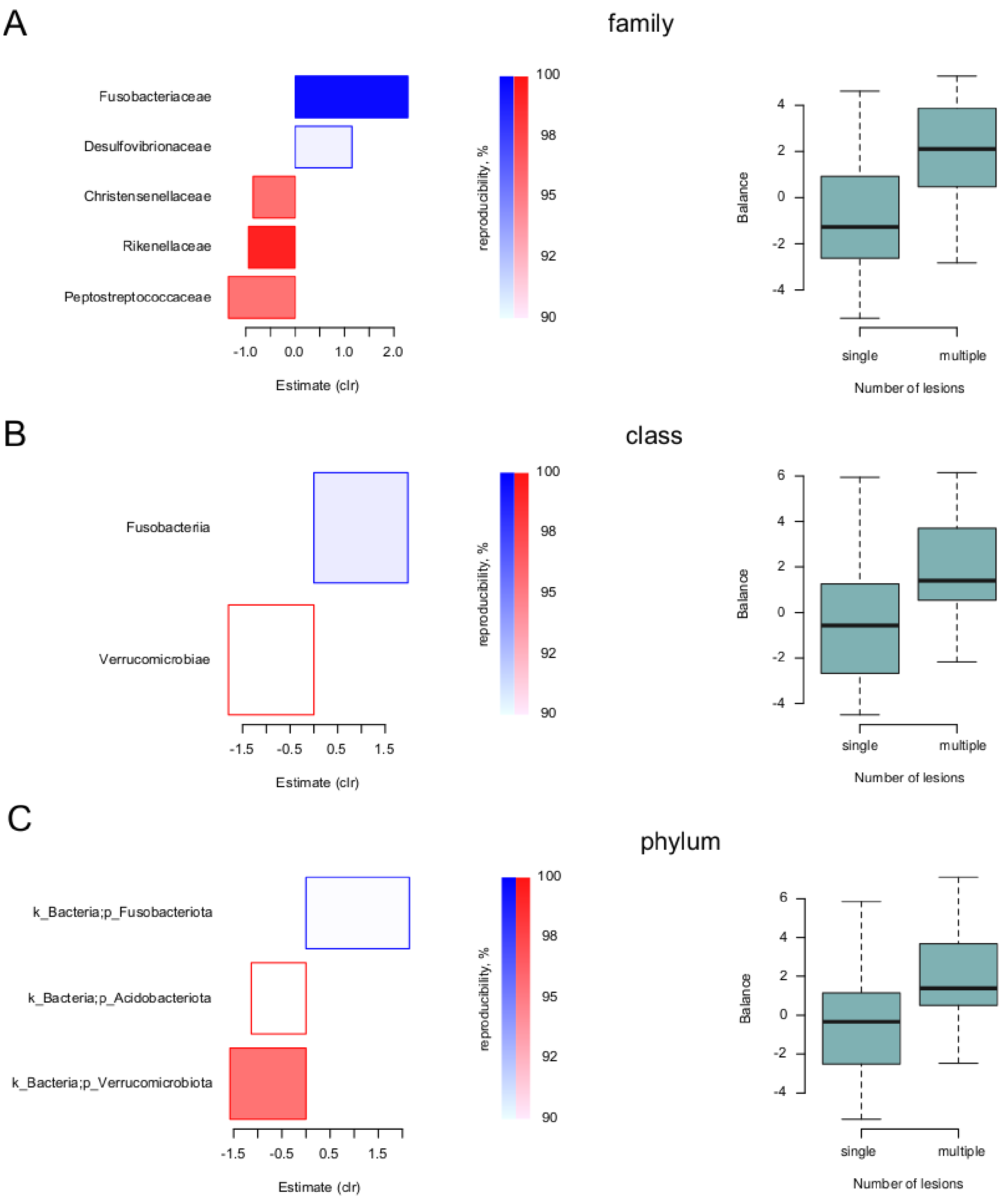

Supplementary figure S1 - Link of number of lesions to tumor-associated microbiome: results of "nearest balance" method for the biopsy samples. On the left: bacterial species - reproducible members of the nearest balances revealed in cross validation. The upper blueish bars show the taxa in the numerator in the order of decreasing linear regression coefficient between clr-transformed microbial abundance and factor (x axis). The lower reddish bars show denominator taxa. The color tint is proportional to the reproducibility of the taxa in cross-validation analysis. On the right: distribution of the balance values for each parameter value. A) Family level. B) Class level. C) Phylum level.

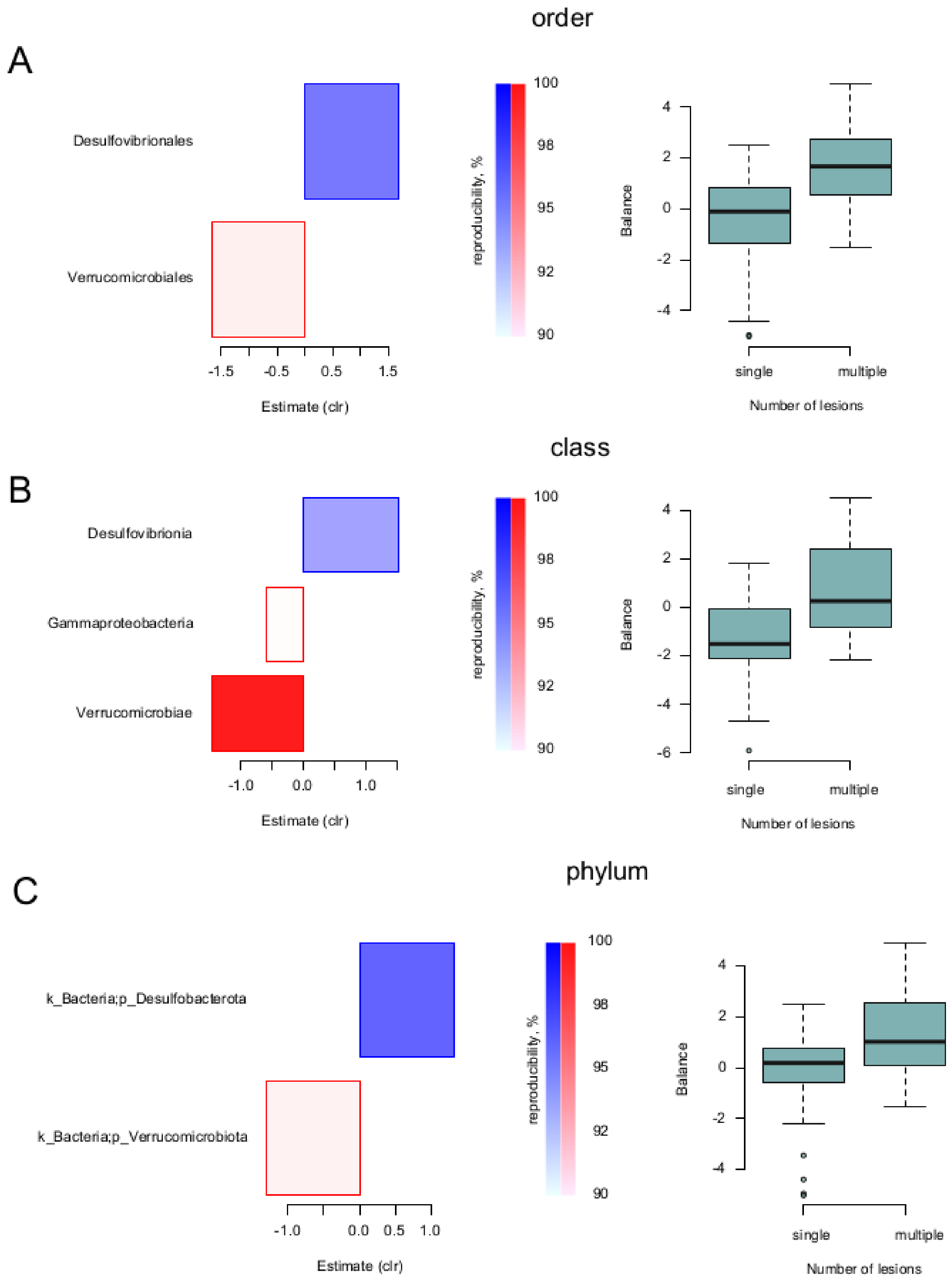

Supplementary figure S2 - Link of number of lesions to luminal microbiome: results of "nearest balance" method for the stool samples. Constructed similarly to Supplementary figure 1. A) Order level. B) Class level. C) Phylum level.

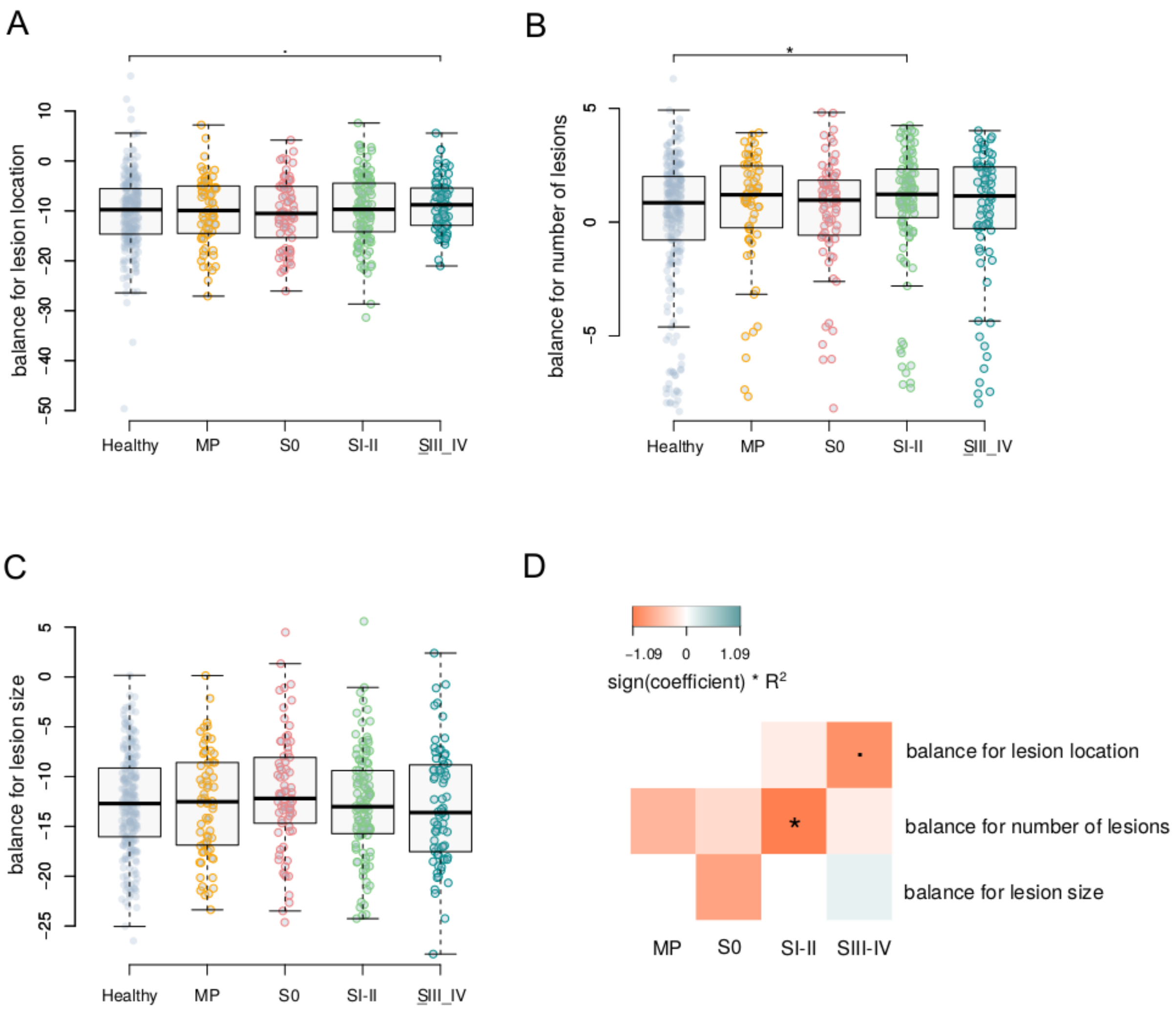

Supplementary figure S3 - Validation of balances associated with precancerous lesions characteristics using public data on stool microbiome of patients with different CRC stages and healthy controls [18]. A-C) Distribution of balances values associated with lesion location (A), number (B) and size (C) in different groups of subjects from public data. D) The result of a linear regression testing the differences in balance values in each group of patients compared to healthy controls. Abbreviations are deciphered in the text. Asterisks denote significant associations (p value<0.05), dots denote suggestive associations (p value<0.1).

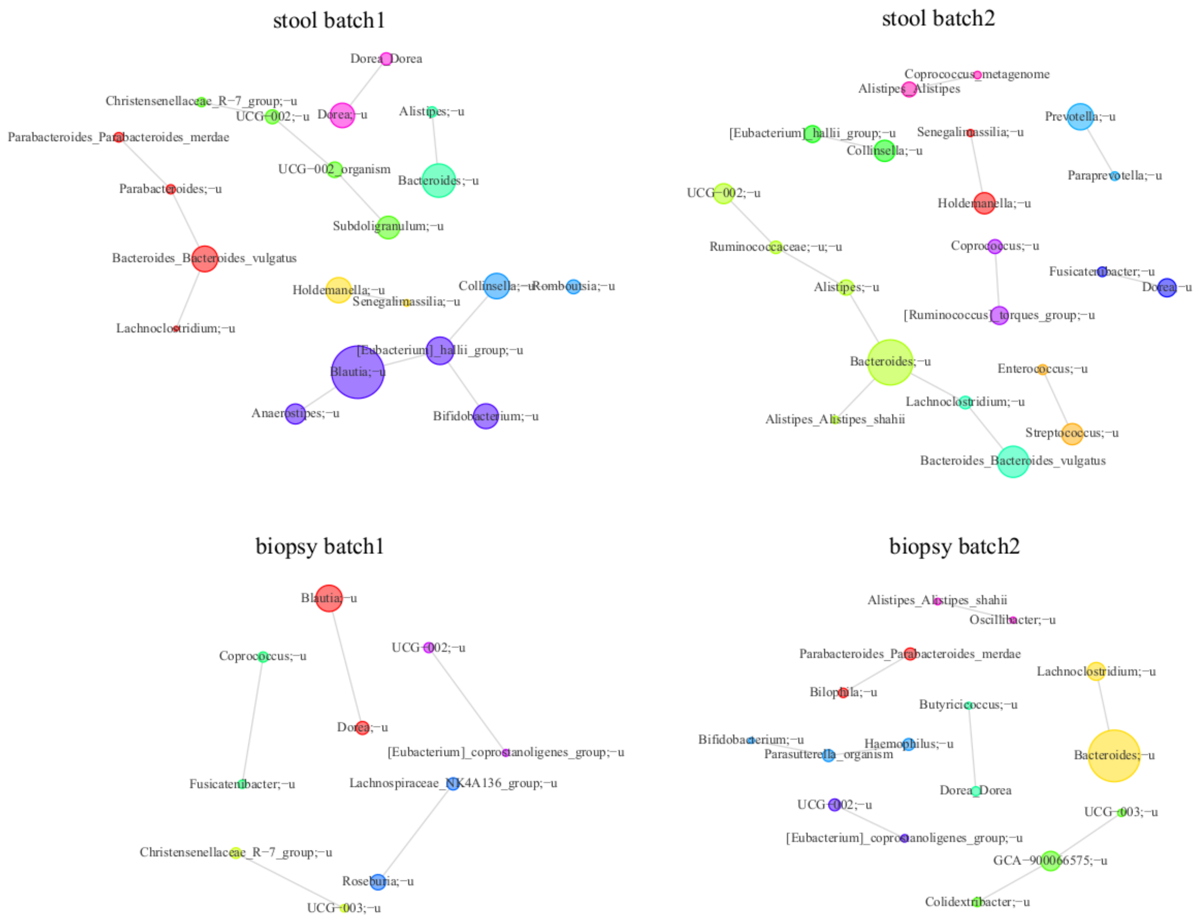

Supplementary figure S4 - Members of microbial cooperatives (co-abundant groups of taxa) obtained for different sample types and batches. Vertex diameter is proportional to the abundance of the taxon. Vertex colors denote different clusters obtained with Louvain method**.**
